# Technological advancements in functional interpretation of genome‐wide association studies (GWAS) findings: bridging the gap to clinical translation

**DOI:** 10.1002/1873-3468.14884

**Published:** 2024-04-29

**Authors:** Redouane Aherrahrou, Minna U Kaikkonen

**Affiliations:** ^1^ A.I. Virtanen Institute for Molecular Sciences University of Eastern Finland Finland; ^2^ Institute for Cardiogenetics Universität zu Lübeck; DZHK (German Centre for Cardiovascular Research), Partner Site Hamburg/Kiel/Lübeck, Germany; University Heart Centre Lübeck, 23562, Lübeck Germany

**Keywords:** CRISPR, eQTL, functional genomics, GWAS, molQTL, MPRA, QTL, single cell sequencing

## Abstract

Genome‐wide association studies (GWAS) significantly advanced our understanding of the genetic underpinnings of diseases. However, challenges persist, particularly in interpreting non‐coding variants in linkage disequilibrium that affect genes in disease‐relevant cells. Addressing key obstacles—identifying causal variants, uncovering target genes, and understanding their network impact—is crucial. This graphical review navigates advanced techniques to fully leverage GWAS for future therapeutic breakthroughs.

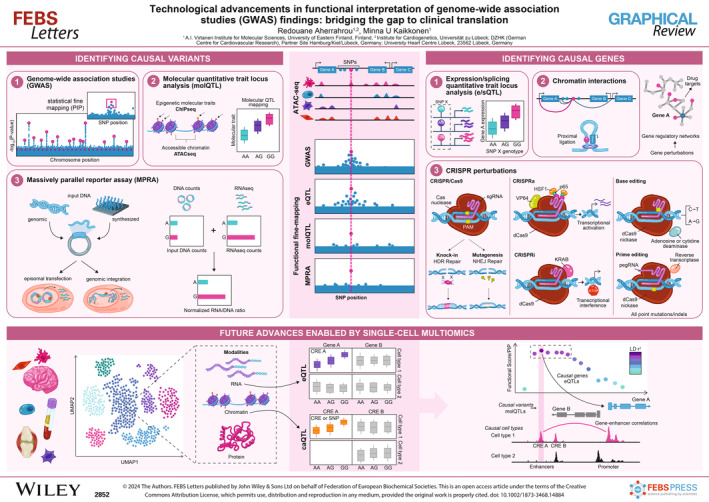

## Graphical abstract

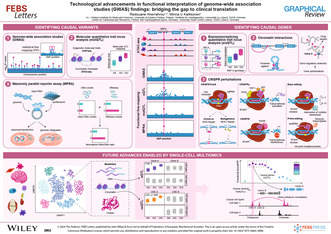

## Main challenges related to GWAS interpretation

Genome‐wide association Studies (GWAS) have led to advances in population and complex‐trait genetics, the biology of diseases (i.e in cancer, diabetes, and cardiovascular diseases), and translation toward new therapeutics [1]. However, the full potential of GWAS has not been unlocked. The primary challenges in interpreting GWAS include identifying causal variants (SNPs), pinpointing target genes, and discerning the subsequent network or pathway changes causing shifts in cellular and physiological roles, and ascertaining the pertinent tissue, cell type, and cell state for all these influences. Variants in strong linkage disequilibrium (LD) with causal variants pose a challenge in discerning between functional and non‐functional variants [2]. Moreover, pinpointing target genes becomes intricate as these functional variants frequently reside in non‐coding cis‐regulatory elements (CREs) associated with disease‐relevant cell types [3]. Such variants hint at their influence on gene regulation (eQTLs) or splicing (sQTLs), necessitating specialized tools, databases and expertise for accurate identification [4, 5]. In this graphical review, we discuss the latest methods that pave the way towards future translation of GWAS findings.

## High‐throughput methods for identifying causal variants

Statistical fine‐mapping is a widely used technique in GWAS data analysis to identify genetic variants directly impacting a particular trait or disease [6, 7]. However, this method encounters several limitations, including the complexity of LD, computational demands, lack of direct functional interpretation, and population‐specific patterns. As a result, experimental methods have gained recognition for their ability to offer direct functional validation of identified variants, providing a more comprehensive understanding of their roles in traits and diseases. In contrast to GWASs largely relying on large population cohorts, these necessitate the use of human disease biobanks that are empowered with omics data. Additionally, they require targeted investigative studies that utilize cellular or animal models, such as zebrafish or mice, to probe deeper into the mechanisms under study.

Over the past decade, the massively parallel reporter assay (MPRA) has gained significant attention for its high‐throughput capabilities, revolutionizing the analysis of numerous variants and regulatory regions [8, 9]. In MPRAs, sequences of CREs and their respective variants are cloned upstream of a minimal promoter that drives the transcription of a reporter gene. Each reporter construct includes a unique barcode or tag. Once cloned, these constructs can be delivered to cells either as episomal entities or integrated directly into the chromosome. Quantification is achieved by comparing the relative frequencies of reads between the DNA library and the RNA expression of the associated barcodes through sequencing. Alternatively, the expression level of the regulatory region itself can be directly assayed using techniques such as STARR‐Seq (Self‐transcribing active regulatory region sequencing) [10, 11]. Although MPRA and STARR‐seq are highly efficient methods to gain a better understanding of CREs in a high‐throughput manner, they have certain limitations. They often rely on artificial reporter constructs, potentially missing the influence of endogenous chromatin architecture and long‐range interactions [12, 13]. Additionally, MPRA may not capture the tissue‐specific or context‐dependent regulatory effects of sequences [13, 14].

The Molecular Quantitative Trait Locus (molQTL) approach is a potent method for exploring the impact of genetic variants on molecular features like chromatin accessibility, histone modifications, DNA methylation, and transcription factor binding within their endogenous genomic context [15, 16]. molQTLs are useful for interpreting the functional roles of non‐coding variants identified in GWAS. By associating genetic variation with changes in molecular traits, molQTLs provide a clearer resolution of potential causal variants, bridging the gap between genotype and phenotype and offering insights into the mechanisms underlying complex traits and diseases. However, very few such studies exist to date, due to the prohibitive costs associated with generating data across large numbers of samples.

## Identifying target genes

Identifying causal genes is a critical step in understanding the biological mechanisms behind complex traits. There are three primary methodologies proposed for assigning target genes to noncoding variants. One of the most widely used methods is eQTL and/or sQTL analysis [4, 5], which correlates variant genotypes with the expression of potential genes or alternative splicing. This is often complemented by colocalization analysis between GWAS and eQTL/sQTL data to determine if the genetic variants associated with a trait are also associated with changes in gene expression or splicing [17, 18]. If the same variant is found to be significant in both, it provides stronger evidence for a potential biological mechanism linking the genetic variant to the trait of interest. Further linking this information to orthogonal molecular, proteomic and/or metabolomic QTLs can provide deeper understanding of the genotype‐phenotype relationship [19, 20]. QTL studies, while informative, face limitations such as population‐specific results, resource‐intensive data generation and difficulty in distinguishing closely linked QTLs within broad chromosomal regions. To address this, complementary experimental techniques based on proximity ligation (e.g. Hi‐C) have been used to map out physical interactions between variant carrying enhancers and target gene promoters [21, 22]. While offering invaluable information about genome structure and function, these techniques also face challenges related to resolution, computational complexity, experimental noise, and the need for complementary approaches.

Recently, the field has been revolutionized by CRISPR genome editing technologies. These have introduced a range of tactics to alter causal variants and local CREs in physiologically pertinent contexts. Traditional CRISPR/Cas9 nuclease introduces specific (Homology‐Directed Repair (HDR)) or random (non‐homologous end joining (NHEJ)) genetic modifications, while CRISPRi/a modulates CRE activity to perturb target gene expression. More technically demanding yet precise interventions, like base and prime editing, enable nucleotide‐specific changes, illuminating the direct effects of variants on gene expression [23]. These tools are frequently utilized *in vitro* with cell cultures, but recent advancements have also expanded their application to animal models (i.e. zebrafish and mice) [24, 25]. Furthermore, perturbation of the candidate gene expression aids in pinpointing their influence on the original phenotype. Single‐cell CRISPR screens, like Perturb‐seq and CROP‐Seq, are particularly potent tools for mapping genotypes to phenotypes by measuring their global impact on gene expression [26]. Recently, these methodologies have scaled up to hundreds and thousands of genes [27], facilitating the analysis of transcriptional networks and phenotypic outcomes for the complete list of predicted target genes associated with GWAS loci.

## Future advances enabled by single‐cell multiomics

The advent of single‐cell genomics has enriched our capabilities within genetics, particularly enhancing the precision of GWAS translation. Traditional GWAS and eQTL methods, relying on effects across mixed cell populations in tissues, often miss nuanced genetic regulatory mechanisms specific to cell types. Single‐cell eQTL (sc‐eQTL) techniques address this gap, illuminating cell‐specific regulatory effects and revealing transient or rare cellular states crucial for disease processes [4, 28]. Furthermore, incorporating Chromatin Accessibility QTLs (caQTLs) into analysis pipelines enables the correlation of genetic variants with alterations in chromatin accessibility at the single‐cell level, thereby facilitating the identification of causal variants [29, 30]. Concurrently, tools combining protein [31], or chromatin accessibility profiling with gene expression analysis offer further detailed insights into how genetic variants shape cellular phenotypes. For example, the latter approach offers a way to correlate changes in chromatin accessibility (from scATAC‐seq) with gene expression patterns (from scRNA‐seq) across single cells to infer potential gene‐enhancer connections [32, 33]. By unraveling cellular heterogeneity in pathological tissues and integrating this knowledge with GWAS findings, we gain a deeper understanding of genetics’ intricate role in disease at the cellular level. Yet further reduction in costs and optimization of methods for statistical analyses is essential to broaden the application of single‐cell technologies in large biobanks in the future [34].

## Challenges and future prospects

Technological advancements in functional genomics are paving the way for the translation of GWAS findings into clinical applications and therapeutic strategies. However, these endeavors will necessitate large‐scale efforts in the future, including meticulous biobank design and substantial financial investment. Even with a clear understanding of biological mechanisms, the journey toward drug development and therapeutic innovations is intricate. It necessitates rigorous preclinical evaluations and clinical trials, underscoring the need for collaborative endeavors within the research community. In addition to identifying potential drug targets, a critical aspect of translating GWAS results into clinical practice involves the development of Polygenic Risk Scores (PRS) to predict an individual's risk of developing diseases [35]. To guarantee the broad applicability of these discoveries and address health disparities, it is crucial to replicate and validate GWAS findings in diverse populations. This ensures the findings are relevant to all groups and that resulting risk prediction models and therapies are effective and accessible for everyone.

### Sources of funding

This work was supported by the University of Eastern Finland (Researcher Fellowship, to R.A), Academy of Finland (Grant No's 333021 and 335973 to M.U.K), European Research Council Horizon 2020 Research and Innovation Programme (Grant No. 802825 to M.U.K), the Finnish Foundation for Cardiovascular Research (to M.U.K and R.A), The Sigrid Juselius Foundation (to M.U.K), German Centre for Cardiovascular Research (DZHK) (to R.A), Junior Investigator Award from Foundation Leducq (to R.A), and Junior Research Cardiovascular Diseases Grant of the CORONA Foundation (Grant No. S0199/10097/2023 to R.A).

## Disclosures

The authors have nothing to disclose.
